# Duplication of the *SOX3* gene in an *SRY*-negative boy with a 46,XX karyotype: a case report and a review of the literature

**DOI:** 10.3389/fgene.2026.1861766

**Published:** 2026-10-07

**Authors:** Júlia Martinková, Hana Řezáčová, Alena Šantavá, Jana Drábová, Miroslav Štolfa, Radek Vodička, Jiřina Zapletalová, Miroslava Balaščaková, Anna Křepelová

**Affiliations:** 1 Department of Biology and Medical Genetics, Second Faculty of Medicine, Charles University in Prague, Motol and Homolka University Hospital, Prague, Czechia; 2 Department of Medical Genetics, University Hospital Olomouc, Olomouc, Czechia; 3 Centre of Molecular Biology and Genetics, Faculty of Medicine, Masaryk University, University Hospital Brno, Brno, Czechia; 4 Department of Pediatrics, Faculty of Medicine, Palacky University and Olomouc University Hospital, Olomouc, Czechia

**Keywords:** 46,XX male syndrome, 46,XX testicular DSD, disorders of sex development, DSD, *SOX3* duplication, *SRY*-negative

## Abstract

46,XX testicular disorder of sex development (DSD), also known as XX male syndrome, is a rare condition where an individual with a 46,XX karyotype develops a male phenotype. In 90% of cases, the syndrome results from the translocation of the *SRY* gene to the X chromosome or an autosome. The remaining 10% of individuals lack the *SRY* gene. We report a case of an *SRY-*negative 3-year-old boy with a 46,XX karyotype exhibiting normal male external genitalia. Array CGH analysis revealed a 723-kb duplication of the Xq27.1q27.2 region, encompassing the *SOX3* gene. The duplication was tandem. An imbalance in X chromosome methylation was observed, with a ratio of 4.5:1. The patient’s mother and grandmother, both with a 46,XX karyotype, were asymptomatic carriers of this duplication. To date, *SOX3* duplication has been documented in 12 *SRY-*negative male patients and one female patient; eleven of these individuals exhibited various disorders of genital development. In contrast, only two previously reported patients displayed normal male genitalia, although associated with infertility. This case, the first reported in the Czech Republic, further supports the hypothesis that *SOX3* overexpression during gonadal development, accompanied by imbalanced X-chromosome inactivation, may induce testis formation, thereby compensating for the absence of the *SRY* gene in *SRY*-negative 46,XX male patients.

## 1 Introduction

The traditional understanding of mammalian sex determination was initially based on the presence or absence of the Y chromosome. The Y chromosome, containing the sex-determining region Y (*SRY* gene, MIM:*480000), was long regarded as the only factor determining male sex. However, recent discoveries have shown that the absence of the Y chromosome does not necessarily result in a female phenotype. Conversely, the presence of the *SRY* gene, even without the entire Y chromosome segment, can still result in a male phenotype (Moalem et al., 2012).

46,XX disorders or differences of sex development (DSD) (Wiesemann et al., 2010) are categorized into three groups: (1) disorders of gonadal development, including ovotesticular DSD (OT-DSD), testicular DSD, and gonadal dysgenesis; (2) androgen excess; and (3) other (Hughes et al., 2006). 46,XX male syndrome, also known as De La Chapelle syndrome, was first documented in a male patient with hypergonadotropic hypogonadism and a 46,XX karyotype (de la Chapelle et al., 1964). According to an international consensus conference in 2005, the condition is now referred to as 46,XX testicular DSD (Lee et al., 2006; Hughes et al., 2006), where an individual with a female karyotype develops a male phenotype (Wu et al., 2014). The syndrome results from the translocation of the *SRY* gene to the X chromosome or an autosome during meiosis in about 90% of cases (*SRY-*positive 46,XX male, MIM:400045). Affected individuals usually display normal male external genitalia, masculinization, and azoospermia. The remaining 10%–20% of patients, who are *SRY-*negative, often present with ambiguous genitalia, including hypospadias, micropenis, or cryptorchidism (Grinspon and Rey, 2016; Li et al., 2014; Vorona et al., 2007). Patients with *SRY*-negative testicular DSD show full testicular development without ovarian tissue. OT-DSD is characterized by the presence of both testicular and ovarian tissue (Wu et al., 2014). Complete masculinization in individuals lacking the *SRY* gene is extremely rare (Zenteno et al., 1997). The overall incidence of 46,XX testicular DSD is estimated at around one in 25,000, including both *SRY-*positive and *SRY-*negative cases; considering the reported 10%–20% frequency of *SRY-*negative cases, the estimated incidence ranges from approximately 1 in 125,000 to 1 in 250,000 (Berglund et al., 2017; de la Chapelle, 1981).

Sex differentiation is regulated by a complex network of molecular signaling pathways that induce the development of the bipotential gonads into testes or ovaries (Grinspon and Rey, 2016). The critical period has been determined to be 6–7 weeks of gestation (Mizuno et al., 2013). The most significant transcription factor in sex differentiation is SRY protein, which plays a pivotal role in the development of the testes from bipotential gonads and the differentiation of Sertoli cells (Ross and Capel, 2005). SRY activates a cascade that upregulates the expression of the *SRY-*related HMG-box-containing gene 9 (*SOX9* gene, MIM:*608160) (Kashimada and Koopman, 2010). *SOX9* expression is increased in *SRY-*positive cells in XY gonads, initiating Sertoli cell differentiation (Eggers et al., 2014). Despite significant progress in molecular methods, the underlying mechanisms remain incompletely elucidated (Mizuno et al., 2013). Nevertheless, several hypotheses may explain the condition. The most common cause is the translocation of the *SRY* gene in *SRY-*positive individuals (Vorona et al., 2007). The pathogenesis of *SRY-*negative 46,XX DSD is more intricate. It may be attributable to rearrangements of autosomal or X-linked DSD genes, overexpression of key testicular pathway genes, or low-level mosaicism of *SRY* or other DSD genes overlooked by routine molecular genetic methods (Li et al., 2014). *SRY-*negative 46,XX DSD patients with testicular tissue have been documented in association with *SOX9, SOX3,* and *SOX10* rearrangements (Grinspon and Rey, 2016; Vetro et al., 2015; Sutton et al., 2011).

In this report, we present a unique case of an *SRY-*negative boy with a 46,XX karyotype who exhibited unexpectedly normal male secondary sex characteristics and normal psychomotor development. This is the first reported case of an *SRY-*negative 46,XX male patient in the Czech Republic. The present article is supported by a comprehensive review of the existing literature regarding *SRY-*negative 46,XX testicular and OT-DSD patients.

## 2 Case report

The patient was born at 39 weeks and 4 days of gestation to non-consanguineous parents following the mother’s fifth pregnancy. The first-trimester combined screening test was positive for a higher risk of trisomy 21 (1:170) (maternal age was 40 years). A prenatal ultrasound scan (US) identified the fetus as male. However, chorionic villus sampling and amniocentesis revealed a 46,XX karyotype, confirming the absence of the Y chromosome. Further DNA analysis excluded the presence of congenital adrenal hyperplasia. At 3 months of age, the boy was examined due to a discrepancy between the prenatal US and postnatal karyotype results. The patient measured 56 cm in length, weighed 5 kg, and had a proportionate stature with no evident deformities. His genital phenotype was male. An endocrinological examination during minipuberty (age 3.5 months) showed testes in the normal position within the scrotum, measuring 2 mL in volume bilaterally. The penis was of normal length, with erections observed during urination. Psychomotor development was normal. Elevated anti-Müllerian hormone (AMH) levels (>21.00 μg/L) indicated normal testicular function. The hormonal profile was as follows: FSH: 1.4 IU/L, LH: 1.2 IU/L, PRL: 401 mIU/L, E2: <37 pmol/L, T: 1.87 nmol/L, DHT: 0.41 nmol/L, 17OH: 2.68 nmol/L, DHEAS: 8.30 μmol/L, and ADIO: <0.10 nmol/L, consistent with minipuberty (Supplementary Material 2). At 2 years of age, the patient measured 82.5 cm and weighed 11.3 kg, with normal psychomotor development. Toy preferences were not yet defined. Genetic testing was performed on the boy at 3 years of age. Both testes remained scrotal (right testis: 11 × 5 mm, left: 12 × 5 mm) with adequate echogenicity and perfusion. Renal and urinary tract US findings were normal; no Müllerian duct derivatives were detected. Height was 91 cm, and weight was 12.1 kg (both at the 10th percentile for his age). Both measurements confirmed that the patient was eutrophic and without abnormalities. Further examinations revealed prepubertal levels of gonadotropins, testosterone (T), and dihydrotestosterone (DHT). At 3.5 years of age, an hCG (Brevactid) test showed a normal response (T increased from <0.24 nmol/L to 9.29 nmol/L; DHT: 0.41 nmol/L; Supplementary Material 2), indicating functional Leydig cells. His current height (99 cm) and weight (14.5 kg) follow a linear growth curve (10th–25th percentile range) based on his parents’ heights (mother: 165 cm; father: 196 cm). Cognitive development and bone age (assessed at 3, 4, and nearly 6 years of age) were normal for chronological age. Clinical photography at almost 6 years of age is presented in Figure 1.

**FIGURE 1:**
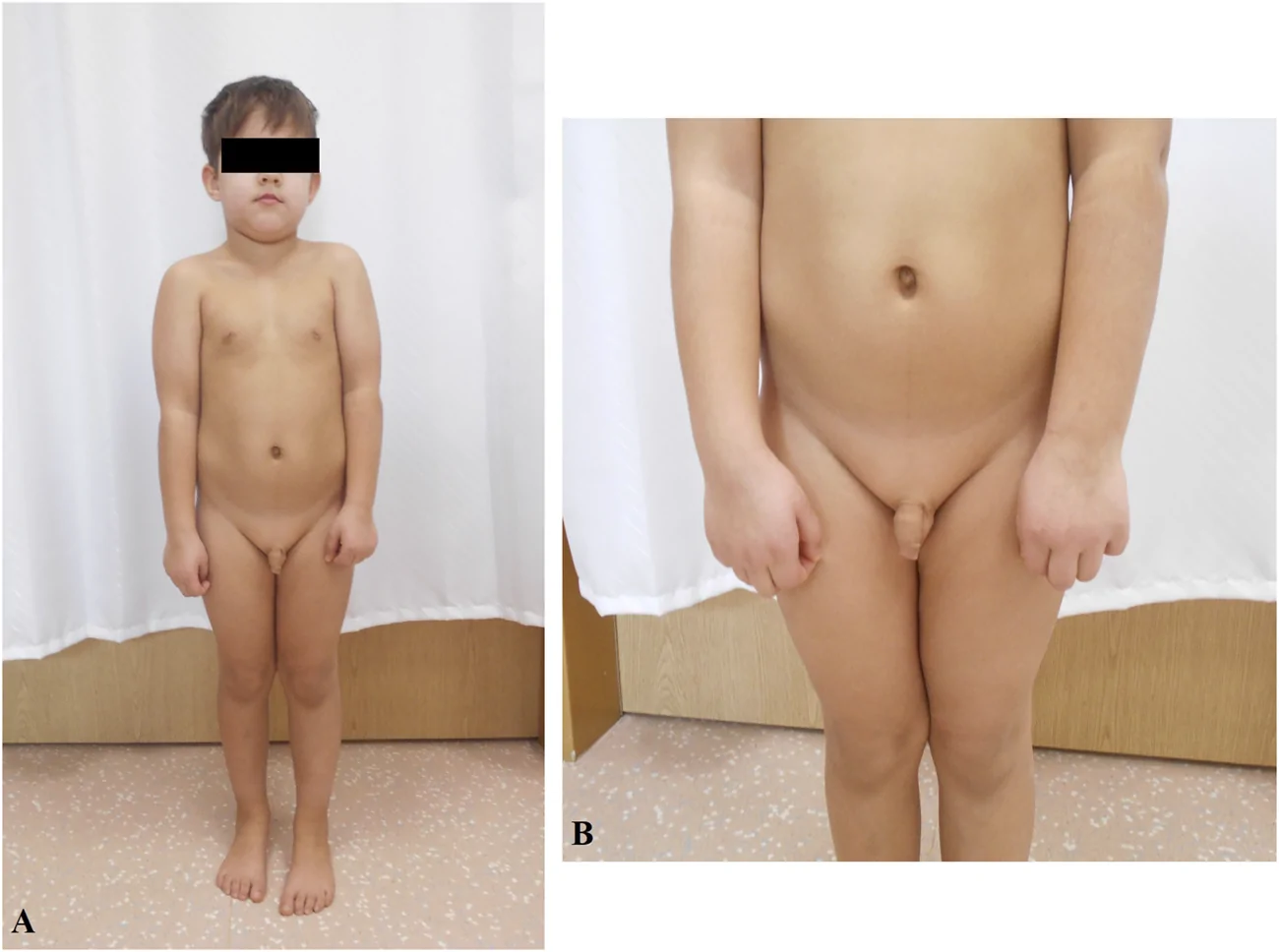
Description of the patient's phenotype with a 46,XX karyotype and *SOX3* duplication. **(A)** Normal proportional stature and non-dysmorphic facies (at approximately 6 years of age). **(B)** Detail of the patient's genitalia. Normally formed male external genitalia with descended testes and a volume corresponding to prepubertal development.

The mother is healthy and has a 46,XX karyotype, with two healthy sisters. One sister has two healthy daughters, and the other has a healthy daughter and son. The first pregnancy of the patient’s mother was terminated due to congenital cystic adenomatoid malformation of the left lung in the fetus; the second resulted in a healthy son (the proband’s 4-years-older brother). Two spontaneous abortions (at 11 and 7 weeks, respectively) preceded this pregnancy. The patient’s father is healthy and has a 46,XY karyotype.

## 3 Materials and methods

### 3.1 Editorial policies and ethical considerations

The Ethics Committee of Motol University Hospital in Prague, Czech Republic, approved the consent form. All procedures were carried out in accordance with the World Medical Association Declaration of Helsinki. The patient underwent molecular genetic and cytogenetic analyses after written informed consent was obtained from the patient’s parent.

### 3.2 Molecular genetic and cytogenetic methods

Initially, karyotyping of the proband was performed using standard G-banding at a resolution of 550 bands per haploid set. Following this, fluorescence *in situ* hybridization (FISH) was conducted on a buccal smear.

The patient’s genomic DNA for molecular genetic analysis was extracted from peripheral blood leukocytes using a standard extraction procedure with the MagCore® Super Automated Nucleic Acid Extractor (RBC Bioscience Corp., New Taipei City, Taiwan). Whole exome sequencing (WES) was performed using the Twist Comprehensive Exome and Twist Mitochondrial DNA panel on a NovaSeq 6000 (Illumina, USA) at the Institute of Applied Biotechnologies a. s. (IAB; Olomouc, Czech Republic) in singleton mode. Single-nucleotide variants (SNVs) were annotated, filtered, and interpreted using the Franklin platform (franklin.genoox.com, Israel) by Genoox. A virtual panel of 198 genes associated with DSD was used to detect pathogenic and likely pathogenic SNVs (see the gene list in the Supplementary File 1). Sequences were aligned to the GRCh37/hg19 reference genome. The pathogenicity of SNVs was assessed according to the guidelines issued by the American College of Medical Genetics and Genomics (ACMG) and the Association for Molecular Pathology (AMP) (Richards et al., 2015). A primary limitation of this study was that the WES, provided by IAB (custom-made), was performed as a singleton assay. Because only one sample was sequenced in the run, copy-number variation (CNV) analysis via WES was not feasible due to the unavailability of parental samples (trio-WES), and a large, uniformly processed reference cohort was needed to normalize baseline variations and ensure analytical sensitivity. To overcome these constraints and accurately detect structural variants, we performed array comparative genomic hybridization (array CGH), which remains the clinical gold standard. Other available technological alternatives for robust CNV resolution include whole-genome sequencing (WGS), which provides uniform genome-wide coverage, and multiplex ligation-dependent probe amplification (MLPA) with custom-made probes for targeted locus validation, although these methods were not utilized in the present study.

Array CGH was conducted using the commercially available oligonucleotide microarray platform SurePrint G3 ISCA 8 × 60 K, V2 (Agilent Technologies, USA), following the manufacturer’s instructions. CNV data were analyzed with CytoGenomics v.5.1.2.1 (Agilent Technologies, USA), which has an average resolution of approximately 200 kb. The clinical significance of deletions and duplications was assessed using the UCSC Genome Browser and the databases DECIPHER, ClinVar, ClinGen, DGV, OMIM, GeneCards, and the GeneScout tool. All coordinates are based on GRCh37. Array CGH was also utilized for segregation analysis in the parents, the patient’s older brother, and the patient’s maternal grandmother.

Furthermore, methylation-sensitive quantitative fluorescent polymerase chain reaction (MS QF-PCR) was performed to determine the patient’s X-chromosome inactivation ratio. The DNA sample was treated with sodium bisulfite and analyzed by MS QF-PCR for the *FMR1* gene, following the modified protocol of Zhou et al. (2006). PCR amplicons were separated and detected by capillary electrophoresis on an ABI PRISM 3130 Genetic Analyzer (Applied Biosystems, Foster City, CA, USA). The relative fluorescence units (RFUs) of the bisulfite-modified DNA amplicons were utilized to evaluate the X-chromosome inactivation ratio. Fragment analysis was performed using GeneMapper software v.4.1 (Applied Biosystems, USA).

Detection of an extra copy of the *SOX3* gene in the proband and his mother was carried out using a custom-made FISH probe (RP11-318H23; Empire Genomics, USA) according to the manufacturer’s instructions.

## 4 Results

Karyotype analysis revealed a chromosomal constitution corresponding to a 46,XX female karyotype. Additionally, FISH confirmed two signals for the X chromosome. DNA analysis confirmed the absence of the *SRY* gene. WES detected no pathogenic or likely pathogenic SNVs associated with DSD genes. Array CGH of the index case revealed two copies of the X chromosome and the complete absence of the Y chromosome. It also identified a 723-kb duplication in the Xq27.1q27.2 region, encompassing three OMIM genes: *SOX3* (MIM:*313430), *SPANXB1* (MIM:*300669), *LDOC1* (MIM:*300402), along with the long intergenic non-protein coding RNA 632 *LINC00632* (MIM:*301147) and microRNA 320days-2 *MIR320D2*. According to the International System for Human Cytogenomic Nomenclature (ISCN) 2024, the patient’s profile was described as: arr [GRCh37] (X)x2 (Y)x0,Xq27.1q27.2 (139584457 × 2,139,584,445_140307864 × 3,140353090 × 2) (Figure 2). Segregation analysis confirmed the maternal origin of the Xq27.1q27.2 duplication via array CGH (data not shown). The maternal grandmother carried the duplicated region, while the patient’s brother did not (data not shown). MS QF-PCR in the index case showed an imbalanced methylation pattern of one X chromosome, with a ratio of 5.77. After correction for preferential amplification of shorter PCR products, according to Vodicka et al. (2008), the true ratio was approximately 4.5 (Figure 3). This confirms skewed X-chromosome inactivation (XCI), favoring the expression of the X chromosome carrying the duplication. FISH analysis of peripheral blood samples from the proband and his mother identified an extra copy of the *SOX3* gene, confirming a tandem duplication (Figure 4).

**FIGURE 2:**
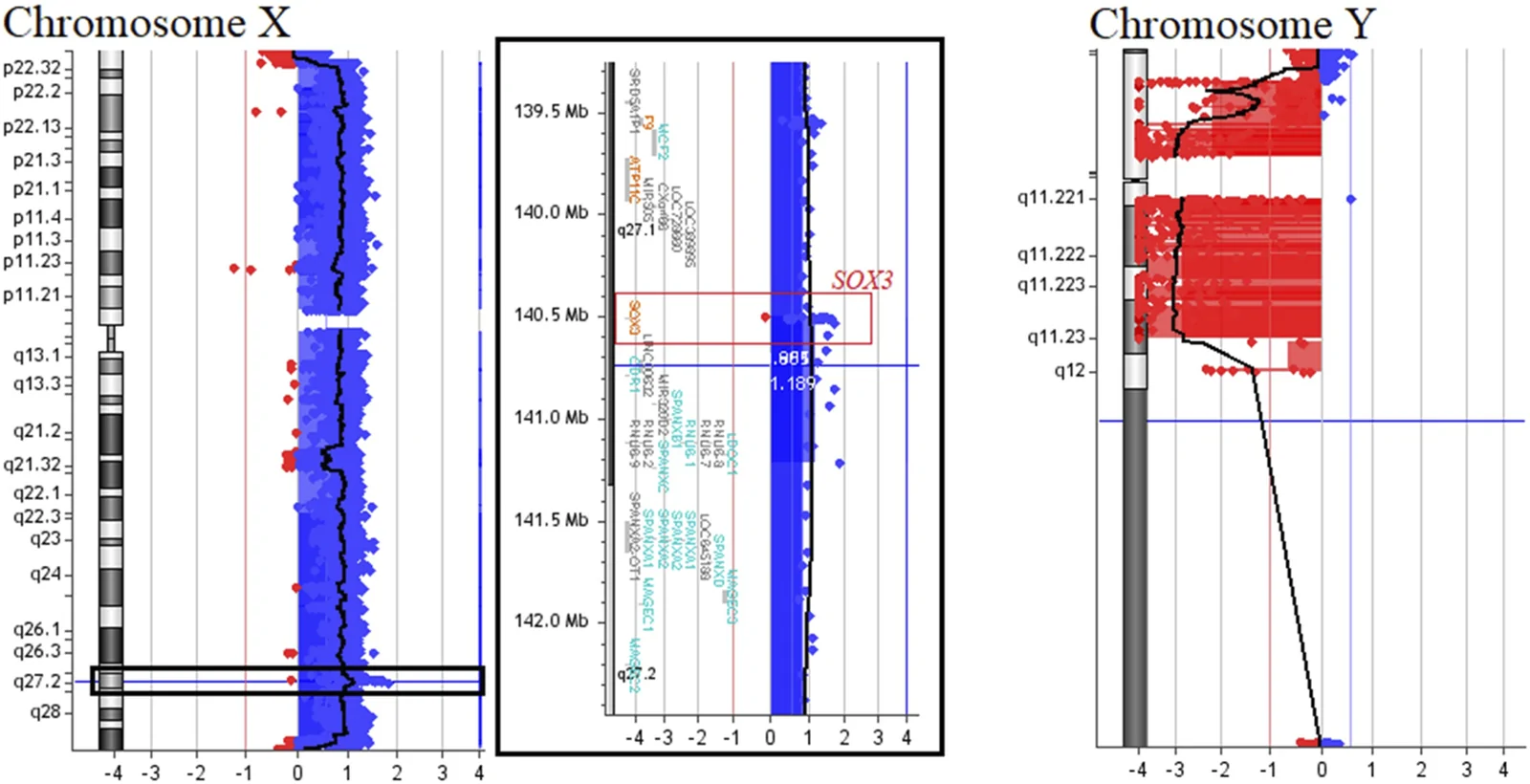
Array CGH results. Array CGH revealed a 723-kb duplication of the Xq27.1q27.2 region, encompassing the *SOX3* gene, in the patient. Red dots represent probes indicating a deletion (value < −0.3), blue dots indicate a duplication (value > +0.3), and the black line denotes a normal copy number (value of 0).

**FIGURE 3:**
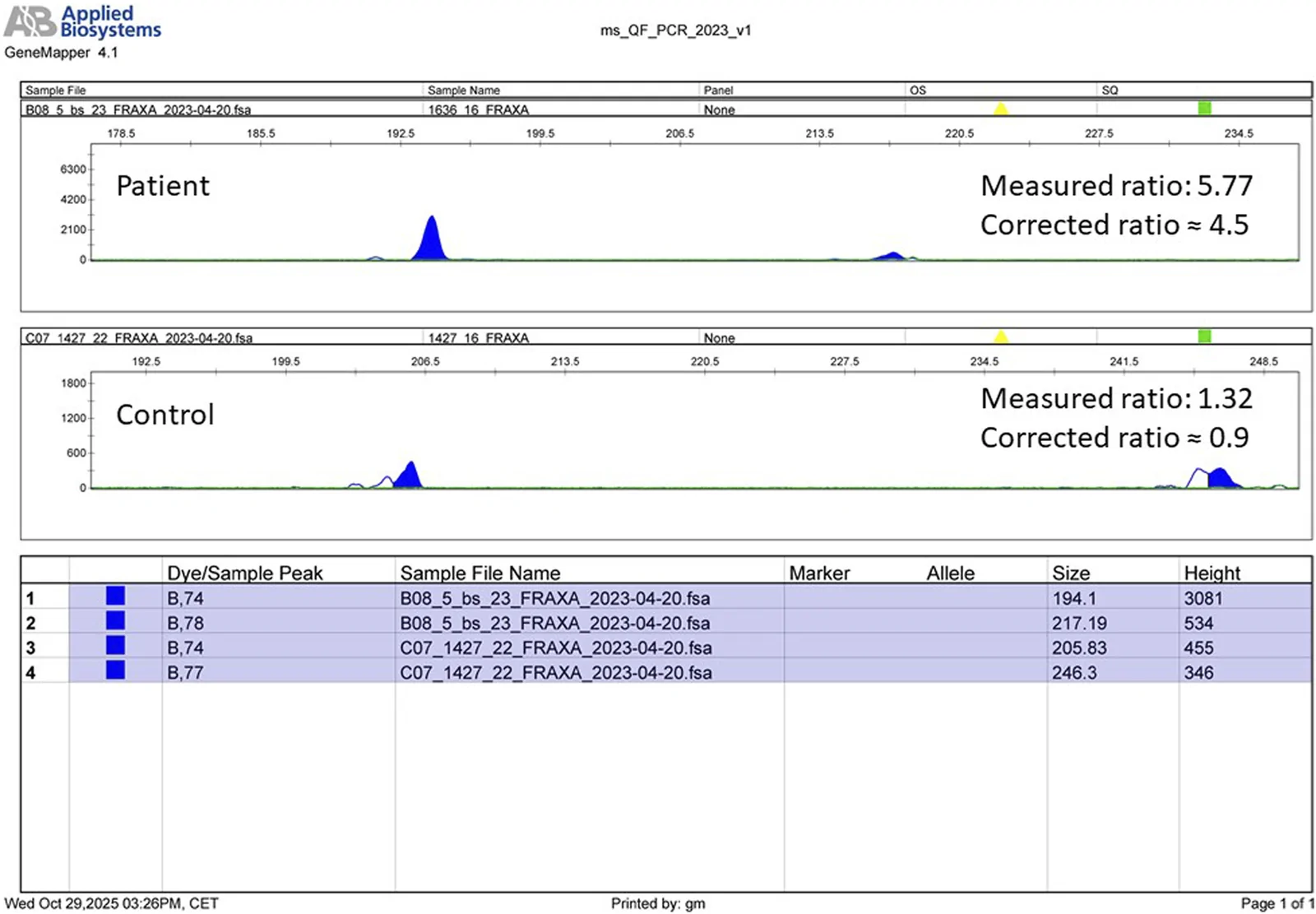
Electropherograms of fluorescently labeled amplicons from bisulfite-treated DNA. Upper panel: Representative electropherogram of fluorescent methylation-specific PCR products from the patient following bisulfite conversion. The amplification peaks represent unmethylated alleles of the X-chromosome (*FMR1* gene). The measured peak height ratio was 5.77 (corrected ratio ≈ 4.5), indicating X-chromosome inactivation (XCI) failure. Lower panel: Corresponding profile for a healthy control, showing a measured ratio of 1.32 (corrected ratio ≈ 0.9). The table below the electropherograms summarizes fragment sizes (bp) and peak heights (RFU) for all detected alleles in both samples. Fragment analysis was performed using GeneMapper 4.1 software (Applied Biosystems). The x-axis represents fragment size (bp), and the y-axis indicates FAM fluorescence intensity (RFU).

**FIGURE 4:**
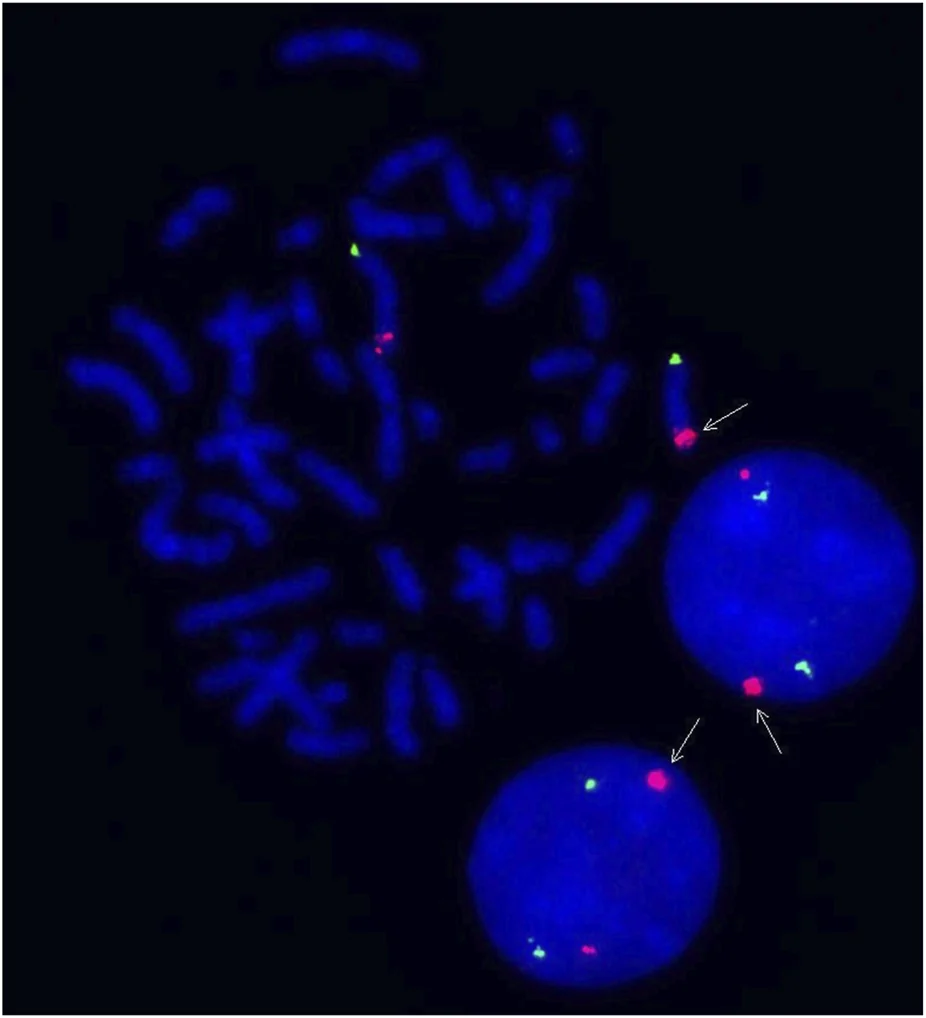
FISH results. FISH analysis demonstrating duplication of the *SOX3* region. The red-labeled probe RP11-318H23 targets the *SOX3* locus (Xq27.1), while the green-labeled probe marks the Xpter as a control. White arrows indicate the duplicated *SOX3* signals.

## 5 Discussion

The *SRY-*related HMG box-containing gene 3 (*SOX3* gene) is homologous to *SRY*. *SOX3* is located in the highly conserved Xq27.1 region and comprises a single coding exon. *SOX3* undergoes X-chromosome inactivation (XCI) and encodes a transcription factor expressed in the central nervous system. It plays a vital role in pituitary, craniofacial, and neuronal development (Grinspon and Rey, 2016; Igarashi et al., 2015; Sutton et al., 2011). Typically, *SOX3* is not transcriptionally active in the bipotential gonad and is therefore not essential for sex determination. However, research in transgenic mice by Sutton et al. (2011) demonstrated that ectopic expression of *SOX3* in developing XX gonads can induce male development by acting as a surrogate for *SRY* (Haines et al., 2015).

SOX3 participates in multiple signaling pathways associated with embryonic development, stem cell proliferation, neurogenesis, and the regulation of cancer progression (Del Puerto et al., 2024). This protein modulates β-catenin activity by interacting with T-cell factor and lymphoid enhancer factors (TCF/LEF) in the Wnt/β-catenin signaling pathway (Kormish et al., 2010). In malignancies such as hepatocellular carcinoma, *SOX3* promotes cell proliferation, migration, and invasion by activating the AKT1-mTOR signaling cascade (Liu et al., 2024). It further impacts NF-κB and MYC pathways (Del Puerto et al., 2024). Post-translational modifications regulate *SOX3* stability, localization, and transcriptional activity, affecting apoptosis, epithelial-mesenchymal transition (EMT), and invasiveness (Del Puerto et al., 2024). For instance, in melanoma cells, SOX3 expression is associated with EMT-related transcriptional changes, downregulating epithelial markers and upregulating mesenchymal markers, thereby enhancing invasiveness (Miranda et al., 2025). During neural differentiation, SOX3 activity is regulated via retinoic acid, highlighting its role in retinoic acid-dependent developmental pathways (Nikcević et al., 2008). Consequently, SOX3 acts as a critical transcriptional regulator in both development and oncogenesis.

Woods et al. (2005) reported duplications of the *SOX3* gene in two brothers diagnosed with X-linked panhypopituitarism (MIM:312000), including growth hormone deficiency, callosal abnormalities, ectopic posterior pituitary, and an absent infundibulum. The duplication was inherited from an unaffected mother with random XCI. The duplicated Xq27.1q27.2 region, encompassing *SOX3*, has been associated with several conditions, including X-linked panhypopituitarism (Woods et al., 2005; Solomon et al., 2004), pituitary hormone deficiency (Rosolowsky et al., 2020), intellectual disability, and neural tube defects such as spina bifida (Butler et al., 2022; Arya et al., 2019; Uguen et al., 2015).

*SOX3* duplication has also been documented in 12 46,XX *SRY-*negative male patients and in one female sibling, as summarized in Supplementary Material 2. Among these patients, eleven exhibited various types of disorders of genital development, including hypospadias (Yang et al., 2025; de Oliveira et al., 2023; Wei et al., 2022; Zhuang et al., 2021; Tasic et al., 2019; Grinspon et al., 2016; Mizuno et al., 2014; Moalem et al., 2012), cryptorchidism (Yang et al., 2025; Oroz et al., 2022; Wei et al., 2022; Zhuang et al., 2021; Grinspon et al., 2016), and bifid or hypoplastic scrotum (Oroz et al., 2022; Moalem et al., 2012; Sutton et al., 2011). Only two individuals exhibited normal male genitalia (Vetro et al., 2015; Sutton et al., 2011), as described below.

All patients in Supplementary Material 2 lacking a Y chromosome are infertile because of the absence of the azoospermia factor (AZF) region (Cioppi et al., 2021). Casas-Vargas et al. (2019) reported an infertile, *SRY-*negative male who had a translocated Y-linked zinc finger protein (ZFY). This factor, located on the Y chromosome, is involved in male sexual differentiation even in the absence of the *SRY* gene. The absence of the Y chromosome probably explains why germ cell cancer is not expected in testicular tissue (Cools et al., 2014), whereas a higher tumor risk is linked to an earlier stage of disruption of Sertoli cell differentiation (Gialouris et al., 2025). This supports the results of a study by Yang et al. (2025), who reported no germ cell tumors or precursor lesions among the 35 *SRY*-negative 46,XX OT-/testicular DSD patients. Gynecomastia is an additional clinical feature in 46,XX male patients. Its prevalence reaches approximately 85% in *SRY*-positive individuals (Zenteno-Ruiz et al., 2001) and 37% in *SRY*-negative individuals (Ergun-Longmire et al., 2005). Clinicians should factor this significant risk into clinical management and provide age-appropriate patient counseling.

Sutton et al. (2011) characterized three *SRY-*negative 46,XX male patients, providing a basis for *SOX3* research. Patient A and C had microduplications involving *SOX3*; notably, the larger rearrangement in Patient C (including 18 additional genes) likely contributed to a more severe phenotype with microcephaly and developmental delay (Supplementary Material 2). Patient B, presenting with testicular dysgenesis, small testes, microcephaly, developmental delay, and gender dysphoria, exhibited a 343-kb microdeletion upstream of *SOX3* (Sutton et al., 2011). Sutton et al. (2011) were also the first to conduct an experimental study in a mouse model, confirming that *SOX3* and *SRY* can synergize with *SF1* to upregulate *SOX9* expression, triggering Sertoli cell differentiation in the bipotential gonad, and thereby inducing sex reversal in XX mice. These data suggest that *SOX3* gain-of-function may act as an *SRY* analog, where increased dosage or ectopic expression triggers the male developmental pathway at a critical stage of differentiation (de Oliveira et al., 2023; Sutton et al., 2011).

Further evidence supporting the role of *SOX3* duplication in 46,XX testicular DSD comes from multiple reports. Moalem et al. (2012) identified a *de novo SOX3* duplication in a patient with penoscrotal hypospadia and a bifid scrotum. Similarly, Mizuno et al. (2014) described four patients with hypospadias, including one with a *SOX3* duplication. Vetro et al. (2015) presented an 8-year-old patient with 46,XX male reversal, mild intellectual disability, and sleep disturbances. The first *SRY-*negative 46,XX OT-DSD cases linked to *SOX3* were reported by Haines et al. (2015) and Grinspon et al. (2016). Haines et al. described a patient with a 774-kb duplication of chromosome 1 inserted distal to *SOX3* (not included in Supplementary Material 2). Concurrently, Grinspon et al. identified a patient presenting with ambiguous genitalia and bilateral ovotestes. Other cases of *SOX3* duplications displayed diverse phenotypes, including atypical genitalia (Zhuang et al., 2021), kidney hypoplasia (Tasic et al., 2019), and prostatic utricles (Wei et al., 2022). Notably, Oroz et al. (2022) employed a custom FISH probe to detect a dislocated non-tandem *SOX3* duplication in a patient with testicular DSD and cryptorchidism. De Oliveira et al. (2023) reported unique siblings with different genders carrying an identical *SOX3* duplication–a female with atypical genitalia and bilateral ovaries, and a male with OT-DSD. Yang et al. (2025) reported an OT-/testicular 46,XX DSD patient with penoscrotal hypospadias, cryptorchidism, and gynecomastia, who underwent partial gonadectomy. As summarized in Supplementary Material 2, these cases substantially enhance our understanding of the mechanisms behind 46,XX DSD. Beyond duplications, other CNVs have been implicated; Sutton et al. (2011) and Qin et al. (2022) reported *SRY-*negative males with microdeletions (343-kb and 867-kb, respectively) in the Xq27.1 region, emphasizing the complex regulatory landscape of *SOX3*.

In this study, we present an *SRY-*negative, 3-year-old male patient diagnosed with 46,XX testicular DSD. Analyses revealed a maternally inherited, 723.4-kb tandem duplication of Xq27.1q27.2 that includes the *SOX3, SPANXB1,* and *LDOC1* genes. Our patient is the third reported case worldwide and the first in the Czech Republic to display normal male external genitalia. A consistent clinical picture (phenotype and genotype) has been previously reported by Sutton et al. (2011) and Vetro et al. (2015).

Most of the *SRY-*negative 46,XX individuals reported to date, including our patient, showed normal psychomotor development in 10 out of 14 (71%) cases, with no signs of intellectual disability (ID) or language delay observed (Supplementary Material 2). Clinical details were lacking for two cases (Yang et al., 2025; Mizuno et al., 2014). However, Sutton et al. (2011) reported developmental delay and growth retardation (Patient C), while Vetro et al. (2015) observed mild developmental delay, mild ID, and language delay.

A *de novo* duplication was identified in seven out of 14 (50%) reported patients (Yang et al., 2025; Wei et al., 2022; Oroz et al., 2022; Zhuang et al., 2021; Grinspon et al., 2016; Vetro et al., 2015; Moalem et al., 2012). The origin of the duplication was not specified by Tasic et al. (2019), Mizuno et al. (2014), and Sutton et al. (2011). De Oliveira et al. (2023) hypothesized germline mosaicism in one parent of two affected siblings. We report a unique Xq27.1q27.2 duplication containing *SOX3* in an *SRY-*negative 46,XX male patient, inherited from his asymptomatic mother and maternal grandmother. Maternal inheritance has also been reported by Woods et al. (2005), although in the context of X-linked panhypopituitarism. In Woods’ patients, a 46,XY karyotype was necessary for phenotypic expression, while 46,XX female carriers remained unaffected (Sutton et al., 2011).

It has been observed that the X chromosome harboring a pathogenic CNV tends to be preferentially inactivated (Igarashi et al., 2015). In the presented case, an imbalanced methylation of one X chromosome (ratio of 4.5:1), confirming skewed XCI, favored the X chromosome carrying the duplication, resulting in 46,XX testicular DSD. The patient’s mother and grandmother were also identified as carriers of the Xq27.1q27.2 duplication; however, they were unavailable for MS QF-PCR analysis to determine X-chromosome inactivation (XCI) status. Although remaining DNA samples from the mother and grandmother were still available, the amount of high-quality DNA after previous sample processing was insufficient for another MS QF-PCR analysis. Since bisulfite conversion required for XCI assessment is associated with considerable DNA degradation and reduced yield, reliable results could not be obtained, and XCI analysis in these family members was therefore not feasible. Subsequently, we lost them to long-term follow-up, and we were unable to contact them for further prospective genetic testing. We hypothesize that their asymptomatic phenotypes may result from skewed XCI favoring the wild-type X chromosome, although we cannot exclude incomplete penetrance or low expressivity of the *SOX3* duplication (Igarashi et al., 2015). Furthermore, the genomic position of the *SOX3* duplication may influence the level of overexpression (Oroz et al., 2022). Following the methodology of Oroz et al. (2022), we employed a custom *SOX3*-specific FISH probe to verify the specific location of the duplication in both the patient and his mother; however, the results showed a tandem arrangement, unlike the findings of Oroz et al. (2022).

Igarashi et al. (2015) documented two duplications: a 230-kb region that includes *SOX3* and a 490-kb region located 44–48 kb upstream of *SOX3* in three individuals: a boy, his mother, and his grandmother. Although the females carried these duplications, they showed random XCI and no 46,XX DSD symptoms. In the boy, *SOX3* overexpression did not affect sex development, although he exhibited Madelung deformity due to *SHOX* overdosage. The random XCI in these females contrasts with previous reports and our current case of *SRY*-negative 46,XX testicular DSD. However, these findings suggest that *SOX3* expression during gonadal development is regulated by multiple *cis*-regulatory elements that can be impacted by CNV alterations or displacement (Igarashi et al., 2015). Moreover, the random XCI observed in leukocyte DNA may not reflect the XCI patterns in the gonads, as these can differ among tissues (Cotton et al., 2011). The XCI ratio of 4.5:1 measured in peripheral blood leukocytes may not accurately represent the embryonic gonadal microenvironment during the critical period of sex determination (six to seven weeks of gestation). Therefore, the normal female phenotypes might be explained by tissue-specific XCI of the chromosome containing the CNV (Igarashi et al., 2015).

Phenotypic variability can also be attributed to the different sizes of the Xq27.1q27.2 duplication, ranging from 123 kb to 6 Mb (Sutton et al., 2011). Our case involved a 723-kb duplication in this region. Specific genes involved and overlapping regulatory regions, such as a 206-kb duplicated segment situated 566 kb upstream of *SOX3*, are sufficient to trigger testicular development in 46,XX individuals (Mizuno et al., 2014). Similarly, a microdeletion upstream of *SOX3* in 46,XX DSD has been reported (Sutton et al., 2011). Further research is needed to clarify the mechanisms behind Xq27.1 rearrangements. Besides *SOX3*, the most frequently duplicated genes in this region include *CDR1* (MIM:*302650) and microRNA 320 days-*MIR320D2*. Our patient exhibited duplications of *SOX3*, *MIR320D2*, and long intergenic non-protein coding RNA 632 *LINC00632*, along with two OMIM genes: *SPANXB1* (MIM:*300669) and *LDOC1* (MIM:*300402) (Supplementary Material 2). Although the roles of these additional genes in 46,XX DSD have historically been unclear, data from genomic databases like ClinGen, Decipher, and GeneCards suggest they may act as important modifiers of the patient’s clinical phenotype.

Specifically, *SPANXB1* and the adjacent *SPANXC* belong to the SPANX family of cancer-testis antigens, which are crucial for spermatogenesis and male germ cell development. These proteins are expressed in postmeiotic spermatids and mature spermatozoa, where they localize to the nucleus and cytoplasmic droplets, participating in sperm maturation and morphogenesis. Although no direct evidence links SPANX alterations to DSD, duplication-driven overexpression could disrupt sperm development, further compounding the primary gonadal failure and infertility expected in this patient (Almanzar et al., 2009; Kouprina et al., 2004; Westbrook et al., 2006).

*LDOC1* encodes a leucine zipper protein that regulates apoptosis and inhibits NF-κB activation (Inoue et al., 2005; Nagasaki et al., 2003). Because NF-κB signaling and apoptosis are essential for cellular differentiation and embryogenesis, altered *LDOC1* dosage could theoretically impair embryonic testicular differentiation and gonadal cord formation. This is supported by MalaCards, which links *LDOC1* dysregulation to urogenital anomalies, including cryptorchidism (Salemi et al., 2011). However, at present, there is no direct experimental or clinical evidence supporting a causative role of *LDOC1* duplications in DSD phenotypes. None of these duplicated genes exhibit known triplosensitivity.

Finally, the duplicated region contains the non-coding elements *LINC00632* and *MIR320D2* (a microRNA). Although these RNAs are known to modulate gene expression and embryonic development, *LINC00632* has primarily been studied in the context of immune regulation and cancer (Statello et al., 2021; Zhang et al., 2019), while altered expression of miR-320 family members is predominantly associated with oncogenesis and metabolic disorders (Rupaimoole and Slack, 2017). While there is no direct evidence linking either element to DSD phenotypes, their co-duplication highlights a complex genomic architecture flanking the SOX3 locus. This altered architecture may disrupt local chromatin structure and regional gene expression regulatory networks. The functional significance of these additional copies in 46,XX DSD remains uncertain and requires further investigation.

Patients with 46,XX testicular DSD and *SOX3* duplications are recommended to undergo testicular surveillance, including scrotal ultrasonography and tumor marker monitoring, even though this risk is considered low in *SRY-*negative individuals (Yang et al., 2025). Additionally, because the lack of a Y chromosome (and consequently the AZF regions) inherently causes absolute azoospermia (Cioppi et al., 2021), early and sensitive fertility counseling is essential to address the biological impossibility of natural conception. This multidisciplinary approach ensures timely oncological screening, while supporting the patient’s psychosocial adjustment and transition to alternative reproductive options.

## 6 Conclusion

*SOX3* duplication has previously been reported in twelve *SRY-*negative male patients and one female patient. Eleven of these patients exhibited various types of disorders of genital development, including hypospadias, cryptorchidism, and a bifid scrotum. Including this report, only three *SRY-*negative 46,XX male patients with normal male external genitalia have been documented. Our case, the first reported in the Czech Republic, further supports the hypothesis that *SOX3* overexpression in developing gonads can induce testicular development in *SRY*-negative 46,XX males. This *SOX3* overexpression likely compensates for the absence of the *SRY* gene, especially in cases of X-chromosome inactivation (XCI) failure. Notably, the patient’s mother and grandmother are also carriers of the *SOX3* duplication but do not exhibit signs of DSD. This suggests that the *SOX3* duplication is necessary but not sufficient to cause DSD, and additional factors likely contribute to the clinical phenotype. Therefore, XCI analysis should be performed in such cases. Our report reinforces the recommendations of Wei et al. (2022) and Tasic et al. (2019) that all *SRY*-negative 46,XX male patients should undergo array CGH to detect *SOX3* rearrangements. Early diagnosis and an understanding of the clinical context are crucial for providing appropriate postnatal care and psychosocial support to patients and their families.

## Data Availability

The datasets presented in this study can be found in online repositories. The names of the repository/repositories and accession number(s) can be found below: https://www.deciphergenomics.org/patient/574503, Patient: 574503.
