# Supplementary material for: Duplication of the SOX3 gene in an SRY-negative boy with a 46,XX karyotype: a case report and a review of the literature
Source: Front Genet. 2026 Oct 7;17:1861766. doi: 10.3389/fgene.2026.1861766 (PMC13683180; doi:10.3389/fgene.2026.1861766)
Supplement: Supplementary file 1 [file Supplementaryfile1.docx]

**Supplementary Material:** Virtual panel of 198 genes associated with disorders of sex development.

*AKAP2, AKR1C1, AKR1C2, AKR1C4, AMELX, AMH, AMHR2, ANOS1, AR, ARL6, ARX, ATF3, ATRX, AZF1, B3GLCT, BBS1, BBS10, BBS12, BBS2, BBS4, BBS5, BBS7, BBS9, BCOR, BMP15, BMP4, BMP7, BMPR1B, BNC2, CACNA1A, CBX2, CCNQ, CDKN1C, CEP41, CHD4, CHD7, CREBBP, CUL4B, CUL7, CYB5A, CYP11A1, CYP11B1, CYP17A1, CYP19A1, CYP21A2, DAZ1, DHCR24, DHCR7, DHH, DHX37, DMRT1, DMRT2, DNMT3B, DUSP6, DYNC1I1, DYNC2H1, EFNB1, EMX2, EPG5, ESCO2, ESR2, EVC, EVC2, FAT4, FBXL4, FEZF1, FGF10, FGF17, FGF8, FGF9, FGFR1, FGFR2, FGFR3, FIG4, FKBP4, FLNA, FLRT3, FOXF2, FOXL2, FRAS1, FREM2, FSHB, FSHR, GATA4, GHR, GLI2, GLI3, GNAS, GNRH1, GNRHR, GPC3, HBA1, HCCS, HDAC8, HESX1, HFE, HFM1, HHAT, HNF1B, HOXA13, HOXA4, HOXB6, HS6ST1, HSD17B2, HSD17B3, HSD17B4, HSD3B2, IL17RD, INSL3, IRF6, KISS1, KISS1R, LEP, LEPR, LHB, LHCGR, LHFPL5, LHX1, LHX3, LHX4, LHX9, LMNA, MAMLD1, MAP3K1, MCM9, MED12, MID1, MKKS, MKS1, MYRF, NEK1, NLGN4X, NMT2, NR0B1, NR2F2, NR3C1, NR5A1, NSMF, NUP107, OPHN1, PAX2, PBX1, PCNT, PCSK1, PDE4D, PEX1, PITX2, POLR3A, POR, PROK2, PROKR2, PROP1, PSMC3IP, PTDSS1, PTPN11, RBBP8, RIPK4, ROR2, RSPO1, RXFP2, SALL1, SEMA3A, SETBP1, SOS1, SOX10, SOX2, SOX3, SOX9, SPECC1L, SPRY4, SRD5A2, SRY, STAR, TAC3, TACR3, TBCE, TBX15, TDRD7, TMEM70, TOE1, TP63, TRAIP, TRIM32, TSPYL1, TTC8, TWIST2, TUBB3, UBR1, WDR11, WDR35, WNT4, WNT5A, WNT7A, WT1, WWOX, ZEB2, ZFPM2, ZFY*
